# Throughput fairness trade-offs for downlink non-orthogonal multiple access systems in 5G networks

**DOI:** 10.1016/j.heliyon.2022.e11265

**Published:** 2022-10-28

**Authors:** Osama Abuajwa, Mardeni Bin Roslee, Zubaida Binti Yusoff, Loo Chuan Lee, Wai Leong Pang

**Affiliations:** Centre for Wireless Technology (CWT), Faculty of Engineering, Multimedia University, Cyberjaya 63100, Malaysia

**Keywords:** Integer linear programming (ILP), Successive interference cancellation (SIC), Penalty function (PF), Particle swarm optimisation (PSO), Jain's fairness index (JFI)

## Abstract

In this work, user pairing and power allocation are proposed as a hybrid scheme to maximise throughput and achieve system fairness in the non-orthogonal multiple access (NOMA) system in 5G networks. The proposed approach is designed to improve the throughput and fairness performance of the downlink NOMA system in 5G networks. User pairing and power allocation schemes are separated to reduce resource allocation complexity. Integer linear programming is applied to perform user pairing, and particle swarm optimisation is used for power allocation. Moreover, the optimisation problem is formulated by converting multi-objective functions into a single function using the scalarisation of multi-objective optimisation problems, and the penalty function is used to prevent optimisation from violating the power, fairness, and data rate constraints. Simulation results show that the proposed model outperforms the conventional numerical approach by at least 9% of throughput maximisation and achieves an acceptable fairness rate.

## 1 Introduction

Non-orthogonal multiple access (NOMA) is a potential access scheme for 5G networks, which multiplexes multiple users on the same radio resources frequency, code, or time with different power levels in the power domain. The NOMA technique exploits the power domain to multiplex multiple users simultaneously, unlike the orthogonal multiple access (OMA). Thus, NOMA can support 5G requirements, such as massive connectivity, enhanced spectral efficiency, and sum-rate, which can support a balanced data rate in the system. In NOMA, the base station (BS) superimposes the message on the same subchannel for multiplexed users via superposition coding, and successive interference cancellation (SIC) is applied at the signal detection receiver [1], [2]. The NOMA system can provide balanced throughput for users in the network in accordance with the channel conditions. A fairness criterion that can be implemented involves the use of a strong channel condition as a relay to improve the data rate for the poor channel condition, which may improve the reception reliability for the poor channel condition user. However, this improvement can result in additional channel resources, such as dedicated time slots or power balance [3]. Hence, the power allocation scheme and user pairing can be applied to support fairness with less system complexity [4].

Power allocation optimisation is formulated as a non-convex optimisation problem, which is difficult to solve directly [5], [6], [7]. Furthermore, power allocation and user pairing are coupled problems, whereas the trade-off between throughput maximisation and fairness is an additional optimisation challenge. Moreover, the analysed work indicates that fairness can be affected by the cell radius, the number of users per cell, and the path loss channel coefficient [8], [9], [10]. The cell radius directly affects the path loss, which depends on the distance between the users and BS. Thus, the number of adopted users is affected by the cell radius, where the user's distance is limited, and the number of users restricts the throughput balance.

For example, a previous study formulated beamforming and power allocation as a joint problem to achieve a fair user data rate by exploiting a maximum–minimum fairness scheme to maximise the minimum achievable user fairness based on the mm-wave NOMA system [8]. The researchers initially obtained the closed-form optimal power allocation from problem formulation and then designed the beamforming vector to reduce joint optimisation [8].

In [9], Xu and Cumanan proposed a simple alternating optimisation scheme to allocate power with *α*-fairness when Karush-Kuhn-Tucker conditions are satisfied for the NOMA system. Al-Wani et al. [11] developed a user selection with a two-stage algorithm using proportional fairness with zero-forcing beamforming to achieve a good trade-off between throughput and fairness for the downlink NOMA system for 5G networks. The proposed scheme focuses on short-term fairness, which involves determining the minimum time window that ensures and evaluates the specified fairness using Jain's fairness index (JFI). In [12], Salehi, Neda, and Majidi developed a finite block length to achieve an optimal power allocation that could also ensure fair throughput for a downlink NOMA system with two users. Their investigation focused on achieving maximum-minimum fairness whilst enhancing the total throughput. The authors in [4] proposed iterative bisection algorithms for power allocation in accordance with the fairness criteria in the downlink NOMA system with instantaneous channel state information (CSI) and average CSI. They proposed that the max–min fairness can be achieved by maximising the minimum user data rate according to the instantaneous channel condition to achieve an efficient bit per channel use.

The authors in [5] divided the resource allocation problem into three subproblems: channel power allocation, power ratio, and two-sided matching. First, the two-sided matching process was applied to match users and channels. Then DC was used to optimise the channel power and the power ratio. After converting the problem to a convex problem using sequential quadratic programming (SQP). The NOMA-DC achieved a near optimal solution for the power allocation problem that is better than NOMA using fractional power allocation (NOMA-FTPA) with a decay factor value of 0.4 [5].

Alternatively, the authors in [13] proposed power and time resource allocation optimisation based on time-sharing to maximise energy efficiency and throughput fairness for downlink time-non-orthogonal multiple access systems using an unmanned aerial vehicle (UAV). The joint optimisation scheme is proposed to exploit the advantages of UAVs in communication systems for the maximisation of throughput fairness and energy efficiency.

Jacob, Panazio, and Abrão in [10] proposed Dinkelbach's method for power allocation and user clustering to achieve equal data rate and maximum energy efficiency for a downlink multiple-input and multiple-output NOMA system. Moreover, the analysed work assumes that fairness can be affected by the cell radius, number of users per cell, and path loss channel coefficient. The authors in [14] developed hybrid beamforming to improve the rate of fairness and power consumption as multi-objectives. The optimisation scheme is used as an inner approximation algorithm and graph theory based on power control and quality of service (QoS). In [15], a fair NOMA scheme is developed on the basis of the scheduling paradigm, considering the idea that the users can always achieve the data rate and compare it if they are using OMA. The authors also derived power allocation coefficient bounds as a channel gain function for two users.

The authors in [16] developed a scheme for a fairness system based on the non-uniform power distribution to measure the rate difference amongst the users to achieve data fairness. User's data rate is measured with a fraction of the total power allocated to the user whilst continuously checking the fairness index, where the value 1 is attained at fair rates. The authors in [17] investigated spectrum resource and power allocation to achieve the minimum required data rate for each user and throughput maximisation as a trade-off scheme for the NOMA system. The work formulated the problem as a double objective optimisation problem; power discretisation was also used to convert the problem into single-objective optimisation (SOO). Moreover, the user-subchannel problem and power allocation are achieved using global optimal search (GOS) to determine the throughput upper bound for each user because of the high complexity. The power allocation scheme considers the adaptive proportional fair for throughput fairness and applies power allocation using classical water-filling based on matching theory (MWF).

## 2 Motivation and contribution

In [6], the proposed scheme is applied as a standalone simulated annealing algorithm for resource allocation to maximise throughput for the NOMA system. The approach can maximise the total throughput, but results in less fair throughput distribution. The resource allocation optimisation schemes can achieve maximum throughput in NOMA, but the low channel gain user may not achieve an acceptable throughput that can balance the total throughput. Moreover, the resource allocation problem is defined as a non-convex problem, where the direct numerical method is required to transform the non-convex problem into a convex problem to achieve an effective solution. However, converting the problem can produce an approximate solution, but no tractable solutions are obtained. Thus, this work aims to maintain throughput maximisation and system fairness without converting the optimisation into a convex problem. Indeed, PSO is proposed to achieve the optimal power allocation without changing the problem, while the near-optimal solution was achieved when the problem was converted from non-convex to convex using DC. The PSO algorithm is applied with simple concepts, controllable parameters, and fast convergence to provide a reasonable solution. Therefore, PSO is applied as a suitable algorithm without converting the problem to achieve better solutions than the near-optimal solutions achieved by DC.

Unlike previous works that converted the optimisation into a convex problem [18], [19], [20], fairness is also studied separately without involving the fairness criteria, minimum user data rate, and power constraint in the objective function. The proposed scheme uses a hybrid scheme (ILPSO) for user pairing and power allocation based on ILP and PSO to achieve trade-offs between throughput maximisation and fairness. ILP is a method proposed to perform the user pairing scheme to reduce resource allocation problem complexity [21]. The proposed scheme formulates the optimisation problem by converting the multi-objective functions into a single function using the scalarisation of multi-objective optimisation (SMOO). The weighted sum method (WSM) is also applied in the design of the SMOO problem. The optimisation techniques used in PSO are based on the behaviour of natural organisms, such as birds flocking and fish schooling [21], [22]. Cooperation amongst individual animals assists the group in reaching its common objectives, such as sourcing food within an efficient time [23], [24], [25].

## 3 Paper organization

The rest of the paper is organised as follows. In section 4, the NOMA system model is described, and the problem formulation for the objective function is proposed. In section 5, the user pairing scheme is proposed, and the PSO algorithm used for power allocation is presented. Section 6 describes the simulations of the proposed algorithms. Finally, section 7 summarises the results.

## 4 NOMA system model

The system considers a single-cell NOMA network equipped with a single-input single-output design, in which the BS is located at the centre of the cell. The number of users and subchannels is defined as *U* and *N*, respectively. The *U* users are uniformly distributed within the cell. The BS transmits the superimposed signal for multiple users over the *N* subchannels, where the subchannel is indexed as n∈{1,2,…,N}. The total bandwidth $B_{\mathrm{Total}}$ is divided by the number of subchannels, and the subchannel bandwidth, $B_{sc}$, is divided equally for each subchannel. Moreover, multiple users share the same subchannel in accordance with the NOMA concepts, where $U_{n}$ is the set of users sharing the same subchannel *n*. The total transmit power distributed over the subchannels is $P_{\mathrm{Max}}$, where $P_{u,n}$ is the user power and system power allocation limited as $\sum_{n}^{N}\sum_{u}^{U}P_{u,n}\leq P_{\mathrm{Max}}$. The BS transmits the signal $x_{n}$ on subchannel *n*, and the transmitted signal is given as seen in (1) [5], [6], [7]:

(1) $$x_{n}=\sum_{u=1}^{U_{n}}\sqrt{P_{u,n}}{\overset{˜}{x}}_{u,n},$$

where ${\overset{˜}{x}}_{u,n}$ is the modelled symbol denoted as ${\overset{˜}{x}}_{u,n}$, and $\sqrt{P_{u,n}}$ is the user's transmit power, while the square root is applied to represent the data signal as amplitude. The signal received at user *u* over subchannel *n* is described in (2) [5], [6], [7].

(2) $$y_{u,n}=g_{u,n}x_{u,n}+Z_{u,n},y_{u,n}=g_{u,n}\sqrt{P_{u,n}}{\overset{˜}{x}}_{u,n}+g_{u,n}\sum_{i=1,i\neq u}^{U_{n}}\sqrt{P_{i,n}}{\overset{˜}{x}}_{i,n}+Z_{i,n},$$

where the channel gain coefficient between the *uth* user and BS is represented as $g_{u,n}=d_{u,n}^{-1}h_{u,n}$, where $d_{u,n}^{-1}$ represents the path loss coefficient. The Rayleigh fading channel gain is $h_{u,n}$. $d_{u,n}$ is the distance between the *uth* user for each channel and BS. $Z_{i,n}$ is the additive white Gaussian noise with zero-mean complex random variable and $\sigma_{n}^{2}$ variance is $Z_{i,n}∼\mathrm{CN}(0,\sigma_{n}^{2})$. The design indicates that the number of users is double the number of channels (U=2N). The data rate on a given subchannel for the *u*th user is described in (3) [5], [6], [7].

(3) $$R_{u,n}=B_{sc}\log_{2}(1+SINR_{u,n}),$$

where the signal-to-interference-noise ratio is given in (4)-(6) [5], [6], [7]:

(4) $$SINR_{u,n}=\frac{p_{u,n}{\left|g_{u,n}\right|}^{2}}{\sigma_{n}^{2}+\sum_{i=1,i\neq u}^{U_{n}}p_{i,n}{\left|g_{u,n}\right|}^{2}},$$

(5) $$=\frac{\frac{p_{u,n}{\left|g_{u,n}\right|}^{2}}{\sigma_{n}^{2}}}{1+\sum_{i=1,i\neq u}^{U_{n}}\frac{p_{i,n}{\left|g_{u,n}\right|}^{2}}{\sigma_{n}^{2}}},$$

(6) $$=\frac{p_{u,n}G_{u,n}}{1+\sum_{i=1,i\neq u}^{U_{n}}p_{i,n}G_{u,n}},$$

where $p_{u,n}$ is the power allocated to the *u*th user on the *n*th subchannel. $g_{u,n}$ is the channel response coefficient on the downlink for the *uth* user on the *n*th subchannel. $G_{u,n}$ is the channel response normalised by noise for the corresponding user. The system design is strictly multiplex with only two users on each channel. For example, the subchannel *n* multiplexes the two users $U_{1,n}$ and $U_{2,n}$ with ${\left|G_{1,n}\right|}^{2}\geq {\left|G_{2,n}\right|}^{2}$, where the power allocation scheme is $P_{1,n}\leq P_{2,n}$, in accordance with the power domain multiplexing protocol in NOMA [26], [27], [28]. In this design, the hypothesise that subchannels are sorted as follows: $\left|G_{1,n}\right|\geq \left|G_{2,n}\right|\geq \cdots \geq \left|G_{U_{n},n}\right|$. Successive interference cancellation is applied on each channel, where the intended signal can be detected on each user. The high channel gain user initially decodes the signal of the low channel gain user and then cancels the interference power and decodes the intended signal. By contrast, the low channel gain user treats the interference power on the same subchannel as noise. This finding is a common NOMA assumption based on SIC. The total throughput is calculated in (7) [5], [6], [7].

(7) $$R=\sum_{u=1}^{U}\sum_{i=1}^{N}B_{sc}\log_{2}\left(1+SINR_{u,n}\right).$$

### 4.1 Problem formulation

Resource allocation involves user pairing, channel power allocation, and power ratio allocation to maximise throughput for the NOMA system in 5G networks. Such methods aim to maximise the throughput with regard to the power allocation and user pairing. The throughput function is described in (8) [6].

(8) $$f(\delta_{n},P_{n})=B_{sc}\sum_{n=1}^{N}\log_{2}\left(1+\delta_{n}P_{n}G_{1,n}\right)+B_{sc}\sum_{n=1}^{N}\log_{2}\left(1+\frac{(1-\delta_{n})P_{n}G_{2,n}}{1+\delta_{n}P_{n}G_{2,n}}\right),$$

where $\delta_{n}={\left[\delta_{1},\delta_{2},\ldots \delta_{N}\right]}^{T}$ and $P_{n}={\left[P_{1},P_{2},\ldots P_{N}\right]}^{T}$ are the power ratio and channel power, respectively. Furthermore, the power ratio for multiplexed users is denoted as $\delta_{n}$ on a subchannel *n* and $P_{n}$ is the power for subchannel *n*. The system is designed to achieve trade-offs between throughput maximisation and system fairness to obtain the optimal solution. The problem is formulated as follows in (9) with the constraints in (10), (11), and (12) [6].

(9) $$\text{Maximise}\;\;f(\delta_{n},P_{n}),$$

(10) $$\text{Subject to}\;\;\;C_{1}:1^{T}P_{n}\leq P_{Max},$$

(11) $$\;C_{2}:P_{n}\geq 0,$$

(12) $$\;C_{3}:0\leq \delta_{n}\leq 1,$$

where $C_{1}$ is the constraint of the total channel power, which is less than the total transmitted power, and $C_{2}$ is the channel power constraint. The power ratio allocation varies between 0 and 1, which is denoted as $C_{3}$. The channel power vector $P_{n}={\left[P_{1},P_{2},\ldots P_{N}\right]}^{T}$ is transformed into a matrix as $1^{T}P_{n}$. The problem design is formulated on the basis of the SMOO approach to convert it into an SOO function [29]. The main objective function is formulated by adding and pre-multiplying each objective with a supplied weight scale [29]. The weight scale is set in proportion to the relative importance of the objective. Moreover, this method prevents the power ratio from reaching 1. In the previous work, the highest value of the power ratio can achieve the maximum of the objective function [6]. These constraints are applied in the objective function to protect the function from violating the constraint. The fairness value is added as a constant that balances the objective function maximisation. For the implementation, the problem is converted to a minimisation problem; thus, so the throughput function is negated. The design of the SMOO problems uses the WSM [29]. Hence, the throughput objective function is normalised and scaled by the number of users; thus, the calculation can be generalised for any number of users. The normalised objective function is written in (13) [29].

(13) $$q=-\frac{f(\delta_{n},P_{n})}{U},$$

where *q* is the normalised value of the fitness function. The cost function must include other constraints because PSO only allows upper/lower bound constraints. Any violation in the sum of power, fairness, and minimum user data rate constraints is added to the cost function. More violation means a higher cost function; thus, if no violation is found, then $p_{⁎}=0$. Penalty functions are presented as follows in (14), (15), and (16) [29].

(14) $$p_{p}=\max(0,1^{T}P_{n}-P_{Max}),$$

(15) $$p_{F}=\max(0,F_{des}-JFI),$$

(16) $$p_{R}=\max(0,R_{\min}-R_{i}),$$

The constraint can add greater difficulty to the optimisation problem; thus, it is an essential step in making the optimisation flexible and less complex. Consequently, the constrained problems are converted into unconstrained ones using an artificial penalty for violating the constraint as a new PF. The minimisation problem is shown below, where $p_{⁎}$ indicates the corresponding penalties for violations. Furthermore, the objective function is developed in (17)–(22) [29].

(17) $$\text{Minimise}\;\;q+p_{p}q_{p}+p_{F}q_{F}+p_{R}q_{R},$$

(18) $$\text{Subject to}\;\;\;C_{1}:1^{T}P_{n}\leq P_{Max},$$

(19) $$\;C_{2}:P_{n}\geq 0,$$

(20) $$\;C_{3}:0\leq \delta_{n}\leq 1,$$

(21) $$\;C_{4}:R_{i}\geq R_{\min},$$

(22) $$\;C_{5}:JFI\geq F_{des},$$

where $q_{p}$ is the power weight constant, $q_{F}$ is the fairness weight constant, and $q_{R}$ is the user data weight constant. The $F_{des}$ is the fairness weight constant that can be predefined in the system; $p_{R}$, $p_{F}$, and $p_{p}$ are the penalty on user's data rate, fairness, and power violation, respectively, and JFI refers to the entire Jain fairness index. The constraint of the minimum user data rate is denoted as $C_{4}$ in $R_{\min}$, and the fairness constraint is denoted as $C_{5}$.

### 4.2 Jain's fairness index

Jain's fairness index is a well-known metric applied in wireless communications to evaluate system's fairness. The efficient resource allocation scheme can provide a flexible system to achieve fair data distribution in the NOMA system [30]. Therefore, the second objective is to generate a balanced data rate as JFI. The JFI is defined in (23) [30].

(23) $$JFI=\frac{\sum_{i=1}^{U}R_{i}}{U\sum_{i=1}^{U}R_{i}^{2}},$$

where $R_{i}$ is the user data rate and *U* is the total number of users. This indicator varies on the basis of the number of users and the achieved throughput. Two users are multiplexed on the same channel as high and low channel gain; thus, ensuring the minimum user data rate is important for users with low channel gain. Thus, fairness is an indicator of the QoS in the NOMA system.

## 5 Resource allocation problem

Resource allocation is a joint optimisation problem based on user pairing and power allocation problem. The optimisation scheme can be decoupled to reduce system design complexity. User pairing and power allocation are addressed separately to reduce system complexity. Consequently, the user pairing scheme is initially formulated using ILP; then, PSO is applied for power allocation optimisation. Finally, the hybrid scheme is defined as ILPSO.

### 5.1 User pairing using integer linear programming

Integer linear programming is proposed to perform the user pairing scheme because user pairing is addressed as a discrete problem [21], [29]. The ILP method is a decision-making scheme that can improve decision quality.

The user pairing problem formulation is described as follows: the first user is selected first on each channel as a high channel gain user, and the second user is then matched to the same channel as a low channel gain. In this problem, user pairing starts by selecting the high channel gain user and then the second user with low channel gain in each channel. The (U×N) gain matrix is denoted as *M*. For example, let *Z* be (U×N) as a binary selection matrix as given in (24) [21], [29].

(24) $$Z_{ij}=\left\{ \begin{array}{cc} 1\; & \text{if user}\;\text{i}\;\text{in channel}\;\text{j}\;\text{is selected}\\ 0\; & \text{otherwise} \end{array}\right.$$

The first objective of user pairing is to select one user from each channel, that is, the sum of the gains is maximised. Therefore, finding a matrix *Z*, such as the sum of the elements of *MZ* is maximised. This objective can be expressed in the following optimisation problem, as seen in (25) [21], [29].

(25) $$\text{Maximise}\;\sum_{i=1}^{U}\sum_{j=1}^{N}M_{ij}Z_{ij},\begin{array}{ccc} \text{Subject to}\; & 1^{T}Z=1,\; & \text{(a)}\\ \; & Z^{1}\leq 1,\; & \text{(b)}\\ \; & Z_{ij}=\left\{0,1\right\},\;i=1,\ldots ,U,\;j=1,\ldots ,N_{sc},\; & \text{(c)} \end{array}$$

where one user is assigned per subchannel as indicated in (a); no duplicated users are assigned per subchannel as indicated in (b), and (c) the matrix is binary for the number of users and subchannels. The newly updated matrix is denoted as the optimal solution $Z^{H}$. After selecting high-gain users, the channels are updated with the first user selection. The second objective of user pairing is to select one user from each channel, that is, the sum of the gains is minimised. Similarly, this can be expressed as the following optimisation problem in (26) [21], [29].

(26) $$\text{Minimise}\;\sum_{i=1}^{U}\sum_{j=1}^{N}M_{ij}Z_{ij},\begin{array}{ccc} \text{Subject to}\; & 1^{T}Z=1,\; & \text{(a)}\\ \; & Z^{1}\leq 1,\; & \text{(b)}\\ \; & Z_{ij}=0,\;\text{if}\;Z_{ij}^{H}=1,\; & \text{(c)}\\ \; & Z_{ij}=\left\{0,1\right\},\;i=1,\ldots ,U,\;j=1,\ldots ,N_{sc},\; & \text{(d)} \end{array}$$

where one user is assigned per subchannel as indicated in (a); no duplicated users are assigned per subchannel as indicated in (b), (c) any element selected in $Z^{H}$ should be cancelled, and (d) the matrix is binary for the number of users and subchannels, denoting the solution to this problem as $Z^{L}$. The final selection matrix is $Z^{⁎}=Z^{H}+Z^{L}$, which contains a pair of users on each channel. This process is encapsulated by a function $S:Z\to R^{2\times N_{sc}}$. This function performs mapping from a permutation number *z* to vectors of sorted gain values in all channels, which is concatenated vertically as given in (27) [21], [29].

(27) $$S(z)=\left[\begin{array}{cccc} G_{1,1}\; & G_{1,2}\; & \cdots \; & G_{1,N_{sc}}\\ G_{2,1}\; & G_{2,2}\; & \cdots \; & G_{2,N_{sc}} \end{array}\right]$$

Given a value of *z*, the design assesses the (ith,jth) element of S(z), which is denoted as $Z_{ij}(z)$, to obtain the gain value of a particular user *i* with either high or low channel gain on a channel *j*, i.e., Gi,j. Considering that the matrix is a binary selection, the users with 1 will correspond to the channel gain of the selected user, and 0 indicates that the channel gain for the corresponding user is not considered.

### 5.2 Particle swarm optimisation for power allocation

Particle swarm optimisation is an algorithm based on natural organisms seeking habitats with sufficient food, such as birds and schooling fish. It is also known as an agent-based algorithm because it uses multiple agents or particles [21], [22]. PSO implementation is simpler than the genetic algorithm (GA) and ant colony algorithm (ACO), which may share some similarities to its optimisation techniques. PSO works on the basis of real-number-randomness and the global communication amongst swarm particles rather than mutations, crossover operators, or pheromones used in GA and ACO. The PSO algorithm is proposed in this work to obtain the optimal power solution in the NOMA system based on the objective function (8).

In particular, each particle's velocity and position are adjusted according to the group information. In PSO, the number of particles, $Q_{P}$, is predefined; $X_{i}$ is the position, and $V_{i}$ is the velocity of each particle. The dimension $D_{S}$ is the search space for potential solutions of optimisation. The quality of the solution is evaluated iteratively for each particle through an objective function. The personal best of the particle is $P_{\mathrm{best}\;i}$ for the obtained objective function value, which is compared with the global best value as $G_{best}$.

Consequently, the position and velocity of each particle are modified along each dimension. The position and velocity are coupled dynamics of a PSO particle. The velocity dynamics are influenced by inertia and cognitive and social components, which in turn influence the position dynamics of the PSO. The position and velocity dynamics are mathematically described in (28) and (29) [21], [22].

(28) $$V^{i}(t)=w(t)V^{i}(t-1)+r_{1}c_{1}(t)\left(X_{pbest}^{i}(t)-X^{i}(t-1)\right)\;+r_{2}c_{2}(t)(X_{gbest}(t)-X^{i}(t-1)),$$

(29) $$X^{i}(t)=X^{i}(t-1)+V^{i}(t),$$

where *i* is the particular PSO particle; $V^{i}(t)$ is the velocity, and $X^{i}(t)$ is the position of the particle along the dimension $d\leq D_{S}$. *w* is the inertia weight; $c_{1}$ and $c_{2}$ are two acceleration factors, which are denoted as non-negative constants, and $r_{1}$ and $r_{2}$ are two uniformly randomised distributed numbers in the range [0,1]. The inertia and acceleration vary in each iteration to improve the PSO performance in accordance with the following parameters, as given in (30) [21], [22].

(30) $$w(t)=W_{\max}-\frac{t\times \left(W_{\max}-W_{\min}\right)}{t_{\max}},$$

where $w_{\max}$ and $w_{\min}$ are the maximum and inertia weight, respectively. The acceleration factor is determined as follows in (31) and (32) [21], [22].

(31) $$c_{1}(t)=c_{1,0}+\frac{t\times \left(c_{1,f}-c_{1,0}\right)}{t_{\max}},$$

(32) $$c_{2}(t)=c_{2,0}+\frac{t\times \left(c_{2,f}-c_{2,0}\right)}{t_{\max}},$$

where $c_{1,f}$ and $c_{1,0}$ represent the initial values, whereas $c_{2,f}$ and $c_{2,0}$ are the iterative final values of $c_{1}$ and $c_{2}$, respectively. The PSO parameters, constants, and values are listed in Table 1 [21], [22].

**Table 1:** PSO design parameter [21], [22].

Parameters	Values
Size of the particle swarm (*Q*_*P*_)	50
Maximum inertia weight (*W*_*max*_)	0.9
Minimum inertia weight (*W*_*min*_)	0.4
Acceleration constants (*c*_1_; *c*_2_)	1.4962
Maximum number of iterations (*t*_*max*_)	600
Maximum velocity (*V*_*max*_)	0.5
Minimum velocity (*V*_*Min*_)	−0.5

**Figure 1:**
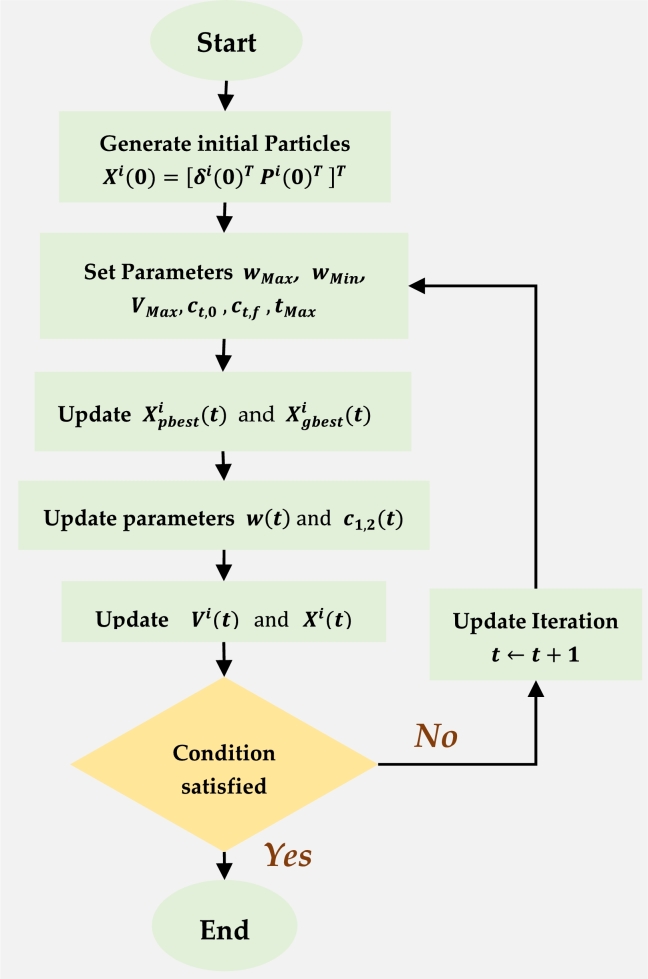
PSO flowchart [21], [22].

The proposed PSO parameters are tuned by trying different values recommended to attain the maximum objective function. The size of the particle swarm is set at least as $Q_{P}=50$, which is sufficient to achieve the maximum objective function with less use of the memory. Moreover, the PSO control parameters achieve less computational efficiency with a smaller number of iterations. The inertia weight values change between minimum as $W_{\min}=0.4$ and maximum as $W_{\max}=0.9$, which influences the velocity of the particles. Similarly, the acceleration constants are set to be equal as $c_{1}=c_{2}=1.4962$. In addition, the maximum and minimum velocity regulation is set between −0.5 and 0.5. The system performance was achieved using the PSO algorithm parameters with about 600 maximum iterations. PSO is used to perform the power optimisation problem by executing PSO as described in Fig. 1 [21], [22].

The optimisation problem is non-convex with regard to variables $\delta_{n}$ and $P_{n}$ for the NOMA system. Therefore, PSO is utilised to obtain the optimal solution as a global optimisation scheme. Let $X^{i}(t)$ be the position vector of a PSO particle *i* in iteration *t*, where $X^{i}(t)$ contains the following optimisation variables as described in (33) [21], [22].

(33) $$X^{i}(t)=\left[\begin{array}{c} \delta_{n}^{i}(t)\\ P_{n}^{i}(t) \end{array}\right]$$

The algorithm generates initial variable particles

(34) $$X^{i}(0)={\left[\delta_{n}^{i}{\left(0\right)}^{T}\;P_{n}^{i}{\left(0\right)}^{T}\right]}^{T},$$

where $P_{n}$ is the subchannel power, and $\delta_{n}$ is the power ratio. The PSO parameters are set as follows: $w_{\mathrm{Max}}$, $w_{\mathrm{Min}}$, $V_{\mathrm{Max}}$, $c_{t,0}$, $c_{t,f}$ and $t_{\mathrm{Max}}$. Then, the objective function is evaluated for each particle as $f(X^{i}(t))$, where $i=1,\ldots ,N_{Particle}$. The possible solution is represented as the position of each particle. Each particle updates the best position $X_{pbest}^{i}(t)$ and then the best position amongst all particles $X_{gbest}(t)$. Subsequently, the inertia weight w(t) and the two acceleration factors $c_{1}$ and $c_{2}$ are updated. Consequently, the velocity and position of a particle are updated until the maximum iterations or the global best $X_{gbest}^{i}(t)$ is reached. This iterative algorithm continues until the optimal power allocation solution is found.

## 6 Result and performance analysis

The NOMA system design was investigated using MATLAB; the simulation parameters are summarised in Table 2. The BS allocates equal bandwidth for each subchannel, where two users are assigned on the same subchannel. The simulation was tested with a minimum number of users of four and a maximum of 20. For simplification, each user was assigned equal bandwidth ($B_{sc,1}=B_{sc,2}=B_{\mathrm{Total}}/N$). The variance $\sigma_{n}^{2}=(B_{\mathrm{Total}}/N)N_{{}^{\circ }}$, where the noise power density is $N_{{}^{\circ }}=-174\;\text{dBm}/\text{Hz}$ [31]. In this design, the radio propagation model includes the distance-dependent path loss with a coefficient of 1 [31], where the shadowing standard deviation is 8 dB [32]. The system design was developed in three scenarios for system performance. The first scenario (ILPSO Scenario 1) was applied without constraint on the minimum data rate and fairness. The second scenario (ILPSO Scenario 2) was applied with only a minimum data rate constraint. Finally, the third scenario (ILPSO Scenario 3) is applied with minimum data rate and fairness constraints.

**Table 2:** System design parameters.

Parameters	Values
Bandwidth (*B*_*Total*_)	5 MHz
Cell Radius	200 m
Maximum Transmitted Power	30 dBm
Noise Power Spectral Density ($N_{{}^{\circ }}$)	−174 dBm/Hz
User Minimum Data Rate	500 b/s
Number of Transmit Antenna	1
Noise Figure	9 dBm
Shadow Standard Deviation	8 dB
Throughput Calculation	Shannon's capacity

**Figure 2:**
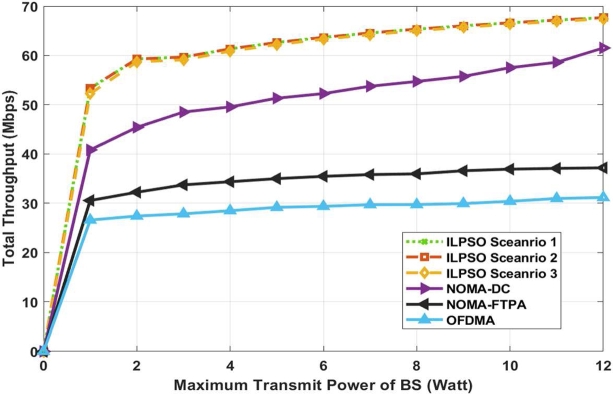
System throughput versus the maximum transmitted power (Watt) for 10 users.

Fig. 2 shows the throughput maximisation versus the maximum transmission power for the NOMA system with 10 users. The proposed algorithm integer linear programming and PSO (ILPSO) outperformed NOMA-DC [5], NOMA-FTPA [33], and orthogonal multiple frequency division multiple access (OFDMA). ILPSO scenarios 1 and 2 outperformed Scenario 3 and the numerical algorithm NOMA-DC, NOMA-FTPA, and OFDMA. ILPSO scenarios 1, 2, and 3 performed user pairing and power allocation, which are 9% better than NOMA-DC and 45% higher than NOMA-FTPA and OFDMA. However, ILPSO scenario 3 still achieves a higher data rate than the existing numerical scheme, whilst considering power constraint, minimum user data rate, and PF. This method aims to prevent the power ratio from reaching the upper and lower power bound, which is mathematically proven in [6].

Furthermore, three scenarios of ILPSO and other existing algorithms show a gradual increment versus the increment of the transmitted power. PSO does not trap in the local optimal solutions, which can achieve an acceptable solution higher than the near-optimal solution for power allocation [5]. Moreover, the numerical algorithm DC [5] was applied by converting the problem from a non-convex to a convex problem, thereby achieving near-optimal power allocation. Therefore, PSO provides acceptable solutions for power allocation that maximise throughput.

**Figure 3:**
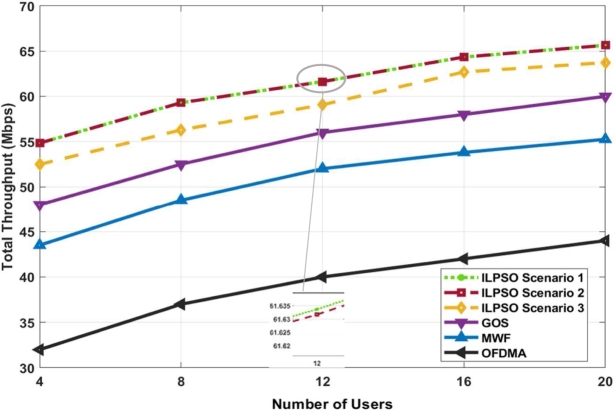
System throughput versus the number of users.

Fig. 3 shows the system throughput maximisation for four to 20 users. The proposed ILPSO outperformed GOS by 9.88%, MWF by 19.5%, and OFDMA by 33% [17]. ILPSO scenarios 1 and 2 outperformed Scenario 3, GOS, MWF, and OFDMA. ILPSO scenarios 1 and 2 achieved higher throughput than scenario 3 because no minimum data-rate and power constraints were applied during the optimisation. However, ILPSO scenario 3 considering the fairness, data rate, and power constraints still achieved a higher data rate than the other schemes. ILPSO scenario 3 showed higher throughput than GOS, MWF, or OFDMA because the system design prioritised fairness more. The system was designed to prevent constraint violation using PF during power optimisation, restricting the power ratio, user data rate, and fairness. Therefore, ILPSO scenario 3 is preferred because it can consider the user data rate, fairness, and power. The three ILPSO scenarios were considered and evaluated in the objective function that achieved better performance than the existing approaches. In addition, the ILP was performed earlier for user pairing based on the common NOMA concepts to relax the problem before power optimisation. Then, PSO was applied for power allocation under two variables restricted by the optimisation penalty to prevent the power constraint from reaching the upper and lower power bounds. The PF and scaled weight were used in the objective function to assist the algorithm in achieving the highest throughput during optimisation.

Moreover, the PSO algorithm was applied in accordance with the predefined user pairing scheme, which achieved a good solution in earlier iterations. The solutions were found after a few iterations were considered reasonable, and the exploration continued to identify all possible solutions and enhance the search for a better solution. By contrast, the power discretisation algorithm [17] was applied by considering the power allocation as a discrete value, where it is a continuous value in wireless communication. Therefore, this power discretisation algorithm may not achieve the highest throughput because it may experience a discretisation error, and more computational complexity is encountered to reduce this error. Hence, the PSO is a metaheuristic algorithm that does not require any algorithm conversion and can easily handle non-convexity and continuous functions without affecting the functional characteristic.

**Figure 4:**
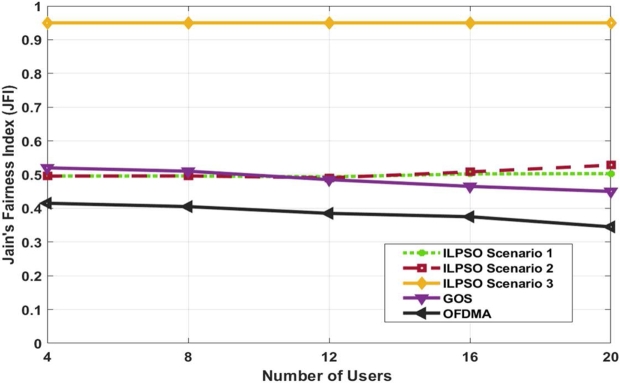
Achieved Jain's Fairness versus the number of users.

As shown in Fig. 4, JFI is evaluated for four to 20 users. The three ILPSO scenarios were compared with GOS [17] and OFDMA. ILPSO scenario 3 shows more than 0.9 of fairness, which is the highest fairness value than ILPSO scenarios 1 and 2, GOS and OFDMA. The superiority of ILPSO scenario 3 is achieved because more weight is assigned to the objective function to prioritise the system's fairness. However, ILPSO scenarios 1 and 2 exhibited a lower fairness value of below 0.5 for the 12 users because the fairness weight was not considered. ILPSO scenarios 1 and 2 showed a slight improvement in fairness for 20 users, whereas ILPSO scenario 2 still achieves better fairness than ILPSO scenario 1. The ILPSO scenarios show better fairness for a greater number of users than GOS and OFDMA. In addition, ILPSO scenario 3 was applied to ensure the system with minimum user data rate and fairness, which showed greater fairness because of the fixed penalty and scaled weight used in the problem formulation. ILPSO scenarios 1 and 2 showed lower fairness values because of the violation of the constraints. Moreover, the ILP scheme was performed on the basis of each subchannel adopting high and low channel gain users with lower complexity than the scheme shown in [17], which achieved user pairing using the exhaustive search in accordance with the scheduling priority based on equivalent channel gain.

**Figure 5:**
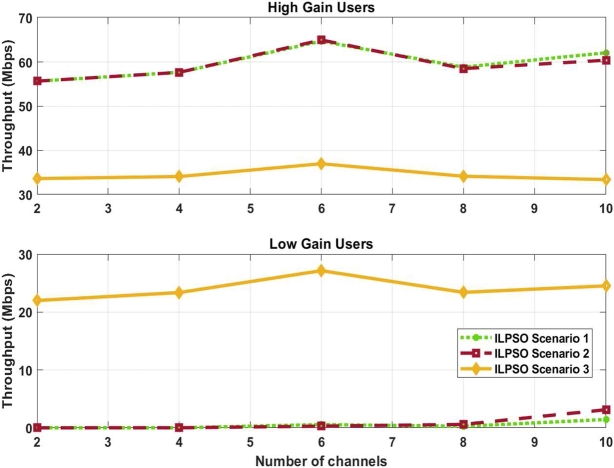
Achieved user throughput for high and low channel gain users.

Fig. 5 shows the user's throughput for a number of channels with a number of users based on the high and low channel gain. The system was designed to ensure that the low channel gain user can still achieve a sufficient data rate because the high channel gain user always achieves a better data rate. Higher gain users showed a high data rate for ILPSO scenarios 1 and 2 and a low data rate for ILPSO scenario 3. By contrast, low channel gain users experience a high user data rate for ILPSO scenario 3, whereas ILPSO scenarios 1 and 2 show a low user data rate. ILPSO scenario 3 shows a better user data rate for low channel gain users than ILPSO scenarios 1 and 2. Hence, ILPSO scenario 3 ensures a better data rate for low channel gain users whilst achieving an acceptable user data rate for high channel gain users. However, ILPSO scenarios 1 and 2 achieve a high user data rate for high channel gain users, but the violation of the constraints causes a user data rate gap between high and low channel gain users. In addition, ILPSO scenarios 1 and 2 cannot provide a good data rate for low channel gain users. ILPSO scenario 2 shows an improved user data rate for the high and low channel gain than ILPSO scenario 1 because of the minimum data rate constraint. Moreover, ILPSO scenario 1 shows a higher user data rate than ILPSO scenario 2 for high channel gain in channel 10 because of the violation of the power and minimum data rate constraint. Consequently, ILPSO scenario 3 achieves a balanced user data rate for high and low channel gain users and ensures a sufficient user data rate for low channel gain users.

**Figure 6:**
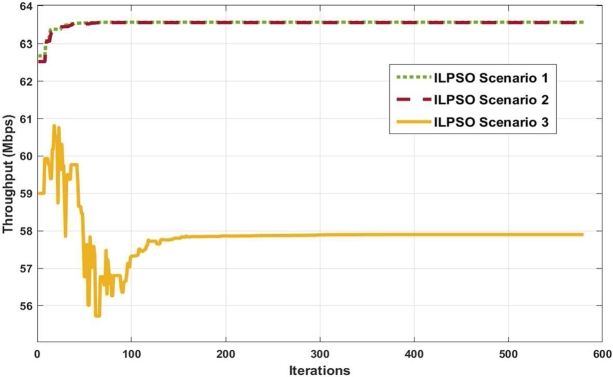
Algorithm convergence versus achieved throughput.

Fig. 6 illustrates the proposed algorithm convergence for the three scenarios with 20 users and 10 channels. ILPSO scenario 3 converges after 150 iterations at lower throughput than ILPSO scenarios 1 and 2, where ILPSO scenario 3 violates the constraints in the first 100 iterations. Moreover, ILPSO scenarios 1 and 2 converge earlier after 50 iterations with higher achieved throughput because of the system design examined without data rate and fairness constraints. The three ILPSO scenarios achieve higher throughput and stop once convergence is reached on the last iterations. Hence, no changes in the convergence through the maximum number of iterations are found; thus, 500 iterations are enough to achieve the acceptable solution. ILPSO scenario 3 converges with at least 200 iterations, whereas 50 iterations are enough for ILPSO scenarios 1 and 2. The ILPSO ended the optimisation as the change per iteration was less than the tolerance value of $1e^{-6}$ for the scenario that finished with a minimum number of iterations. ILPSO runs the risk of producing a sparse solution on earlier iterations, as observed in ILPSO scenario 3. Hence, increasing the swarm size to capture the global optimal solution is unnecessary where the set of constraints is sufficient for the system design. The set of constraints and PF create a flexible system design and smooth the convergence of the ILPSO.

## 7 Conclusion

In this paper, the ILPSO scheme is proposed with three scenarios to optimise resource allocation and achieve trade-offs between throughput maximisation and system fairness in the 5G network based on the downlink NOMA system. Resource allocation complexity can be reduced by separating the user pairing and power allocation schemes. In addition, the ILP was applied for user pairing in accordance with the NOMA concepts, and then PSO was applied for power allocation without considering non-convexity conversion. The WSM was applied to the optimisation problem in the SMOO design to convert it into an SOO function using the PF. The three ILPSO scenarios performed user pairing and power allocation that were 9% better than NOMA-DC and 45% higher than the NOMA-FTPA and OFDMA. Furthermore, the three ILPSO scenarios were investigated to evaluate system's fairness for the cost of the throughput maximisation. The proposed scheme outperformed other work, and ILPSO scenario 3 outperformed GOS by 9.88%, MWF by 19.5%, and OFDMA by 33%. Moreover, the system shows greater fairness than existing schemes, with ILPSO scenario 3 outperforming scenarios 1 and 2. Finally, the proposed scheme can ensure a balanced throughput for users with high and low channel gain and achieve the suitable NOMA system design for 5G network requirements.

## Declarations

### Author contribution statement

Osama Abuajwa: Conceived and designed the experiments; Performed the experiments; Analysed and interpreted the data; Contributed reagents, materials, analysis tools or data; Wrote the paper.

Mardeni Bin Roslee, Zubaida Binti Yusoff: Conceived and designed the experiments; Performed the experiments; Analysed and interpreted the data; Contributed reagents, materials, analysis tools or data.

Lee Loo Chuan, Pang Wai Leong: Analysed and interpreted the data; Contributed reagents, materials, analysis tools or data.

### Funding statement

This work was supported by the MMU-Tel U Join Research Grant (MMU/RMC-PL/TELKOM/AL/030), Telkom Universitas, Indonesia. Osama, Abuajwa was supported by 10.13039/501100003093Ministry of Higher Education, Malaysia (FRGS/1/2017/ICT03/MMU/02/3).

### Data availability statement

The authors do not have permission to share data.

### Declaration of interests statement

The authors declare no conflict of interest.

### Additional information

No additional information is available for this paper.
